# “It’s Not Like My Old Life, But It’s Still Freedom”: An ACTION learning network mixed methods analysis of the adolescent ventricular assist device experience at home

**DOI:** 10.1016/j.jhlto.2026.100685

**Published:** 2026-09-16

**Authors:** Kelly E. Rea, Eleanor Greiner, Katie Flaspohler, Mary C. Jenkins, Dean S. Karahalios, Michelle Mijon, Shannon Oliver, Zachary Hutchinson, Anna Joong, Joseph Spinner, Melissa K. Cousino

**Affiliations:** a Division of Pediatric Psychology, Department of Pediatrics, C.S. Mott Children’s Hospital, Congenital Heart Center, Michigan Medicine, University of Michigan, 1540 E. Hospital Drive, Ann Arbor, MI 48109; b The Heart Institute, Cincinnati Children’s Hospital Medical Center, University of Cincinnati College of Medicine, 3333 Burnet Avenue, Cincinnati, OH 45229; c Baylor College of Medicine / Texas Children’s Hospital, 6621 Fannin Street, Houston, TX 77030; d Division of Pediatric Cardiology, Department of Pediatrics, Ann & Robert H. Lurie Children’s Hospital of Chicago, 225 E. Chicago Avenue, Chicago, IL 60611; e Joe DiMaggio Children’s Hospital, 1005 Joe DiMaggio Drive, Hollywood, FL 33021; f Department of Pediatrics, University of Alberta, 11405 87 Avenue NW, Edmonton, Alberta T6G 1C9, Canada; g Seattle Children's Hospital, Seattle, WA

**Keywords:** adolescent, young adult, ventricular assist device, quality of life, psychosocial

## Abstract

**Background:**

Pediatric patients supported with durable ventricular assist devices (VAD) are increasingly discharged home on device. Little is known about adolescent and young adult (AYA) experiences during this unique time. This study aims to further understand AYA VAD experience to improve supports and interventions upon discharge.

**Methods:**

An 8-domain, 34-item semi-structured interview guide was developed, informed by prior adult VAD interview, pediatric literature, and multidisciplinary VAD team review, including patient/family advocates. AYAs currently or recently discharged home on durable VAD at an ACTION site were approached. Two authors independently coded qualitative responses using a priori codes for thematic analysis. Inter-rater reliability was achieved.

**Results:**

Seventeen AYAs (M=17.40 years) completed semi-structured interviews. The majority were male (76%) and half were White race (53%). All were supported on HeartMate3, most commonly with underlying diagnosis of dilated cardiomyopathy (76%). Length of heart failure ranged from 2 weeks to 9 years prior to device implant. AYAs provided insights across several domains. Most common worries were emergency situations (82%), infection (71%), and bleeding (71%). All reported some limitations, including engagement with friends (71%), sports (59%), and freedom of movement (47%).

**Conclusion:**

Significant life disruptions and coping challenges are prevalent among AYAs discharged home on VAD. Robust psychosocial supports are indicated, including access to multidisciplinary care providers who are aware of the intricacies of managing VAD outpatient. Given remarks from AYAs regarding importance of peer connection, results highlight AYA peer and caregiver support groups and online communities as future directions.

## Introduction

Pediatric patients with heart failure supported with durable ventricular assist devices (VAD) are increasingly discharged home on device. Despite increased durable VAD use and discharge home, little is known about adolescent and young adult (AYA) experience during this unique time. A recent systematic review regarding the pediatric patient and family experience on VAD noted few studies among AYAs.^1^ Findings highlighted AYA hesitancy for discharge home and feeling unprepared for the physical experience of VAD support.^1^ Family experiences centered on adapting to the new VAD lifestyle, navigating many unknowns, and the persistent threat of mortality.^2^, ^3^

Recent studies have sought to further understand psychosocial outcomes among pediatric patients supported with VADs. Pediatric patients on VAD self-report quality of life (QOL) scores lower than pediatric patients in heart failure, heart transplant recipients, and healthy controls.^4^ Further, QOL scores significantly improved at 3 and 6-month post-VAD implant as compared to pre-implant scores, suggesting additional shifts in coping and improved functioning over time.^5^ In the immediate post-implant period (i.e., within 30 days), the most common coping challenges included activity limitations, dressing changes, and pain.^6^ However, as patients were further removed from VAD implant (i.e., 31–100 days post-implant), challenges shifted toward psychological and fatigue-related symptoms, with the most common symptoms being poor sleep, sadness, fatigue, and dressing change distress.^6^ While limitations on activity were prevalent, many pediatric patients also report high levels of happiness.^5^ A recent study among older adults supported outpatient with VAD identified several user experience factors influencing coping, including navigating power supply, adjustments to support wearability and mobility, and handling emergency situations.^7^

As practice evolves beyond VAD as bridge to transplantation to include evaluation for myocardial recovery on device and consideration of chronic device therapy and net-prolongation of life,^8^ long-term outpatient VAD support will become more commonplace for AYAs. While understanding of the impacts of VAD support on psychosocial wellbeing has advanced, less is known about navigating VAD during the unique AYA period. Given the multi-faceted and evolving experience for pediatric patients and families supported on VAD at home, the aim of this study is to investigate the AYA outpatient VAD experience to inform and enhance supports. This work also has the potential to strengthen therapeutic relationships, enhance understanding of nonadherence often observed in this age group, and inform future device development.

## Method

### Study design

This is a multicenter, cross-sectional study of AYA patients supported with a VAD in the outpatient setting. All participants received care at a participating Advanced Cardiac Therapies Improving Outcomes Network (ACTION) site. ACTION is an international learning health network aimed at improving outcomes in children with heart failure.^9^ The protocol was approved by ACTION’s centralized IRB at Cincinnati Children’s Hospital, which houses the data coordinating center.

### Interview guide development

Two authors (KR, EG) independently conducted a literature search focused on peer-reviewed publications from the past 10 years addressing the outpatient experience of pediatric VAD patients and caregivers. Ten resulting articles were reviewed and resulting domains included Discharge Home on VAD, Annoyances & Worries, Sleep, Activities & Limitations, Independence & Responsibility, Resilience, Coping, and Lessons Learned.

The final 8-domain, 34-item semi-structured interview guide was modeled after the previously published adult LVAD survey of user experience by Schlöglhofer and colleagues.^7^ The guide includes multiple choice, Likert scale and free response questions. The interview guide was reviewed by an interdisciplinary team composed of a VAD coordinator, a young adult currently on VAD support, VAD family advocates, and VAD providers (cardiologists and psychologists). The final interview guide is available in Appendix A.

### Study population

Interviews were offered to individuals ages 12–22 years old who, within the past 3 years, were supported on a durable VAD at an ACTION participating site and subsequently discharged home on device. Recruitment was limited to those ACTION sites who had an ACTION cardiology/psychology fellow and/or VAD team member participating in this project. Patients supported on VAD without being discharged home on VAD therapy were excluded, as well as patients who would not be able to complete interview independently due to language or cognitive barriers. All participants previously consented to take part in ACTION data collection.

### Interview administration

Eligible patients were invited to participate in a semi-structured interview led by ACTION cardiology/psychology fellows and/or VAD team members from December 2024 through July 2025. Interviews were approximately 20–30 min in length and conducted virtually via secure telehealth software or in-person based on availability. Responses were documented as close to verbatim as possible in a secure REDCap database hosted at Cincinnati Children’s Hospital.^10^, ^11^ Interviews were not recorded. Participants were mailed a $10 gift card as compensation.

### Analytic plan

For quantitative variables, descriptive statistics were analyzed with SPSS software, version 27.^12^ Qualitative responses were analyzed using Dedoose Version 9.0.107,^13^ a cloud application for managing, analyzing, and presenting qualitative and mixed method research data. Two authors (KR, EG) independently thematically coded qualitative responses by participants using a priori codes. An a priori codebook was developed using prior literature review and established interview guide domain sections. Inter-rater reliability was established based on guideline of > 80% agreement across coders.^14^ There were no significant disagreements across coders therefore consensus was achieved.

## Results

### Demographics

Seventeen AYAs (*M=*17.40 years; range 12–22 years) completed interviews, representing a 36% participation rate across all AYAs supported on VAD and discharged home during eligible study time period at participating sites. The majority were male (76%), approximately half were White race (53%). All participants were supported on HeartMate 3, with majority having an underlying diagnosis of dilated cardiomyopathy (76%). Length of heart failure ranged from 2 weeks to 9 years prior to device implantation and length of support on VAD ranged from 62 days to 859 days (*M* = 303.1, *SD* = 239.2 days). Seven participants (41%) were supported on VAD at the time of interview. Of the ten not supported on device at interview, the majority (90%) were bridged to transplant (*M* time since transplant at interview = 409.8 days, *SD* = 298.0). See Table 1 for full demographic information and medical characteristics.

**Table 1:** Patient Demographics and Medical Characteristics

*N* = 17	*M*(SD), *n*(%)	Range
Age	17.4 (2.6)	12 – 22 years
Gender		
Male	13 (76%)	
Female	4 (24%)	
Race		
White	9 (53%)	
Black/African American	7 (41%)	
Asian	--	
American Indian/Alaska Native	--	
Native Hawaiian/Other Pacific Islander	--	
Mixed race	1 (6%)	
Ethnicity		
Hispanic or Latinx	1 (6%)	
Non-Hispanic or Latinx	16 (94%)	
VAD system		
Heart Mate 3	17 (100%)	
VAD status at time of interview		
Supported on VAD	7 (41%)	
Bridged to transplant	9 (53%)	
Bridged to recovery	1 (6%)	
Underlying cardiac diagnosis		
Dilated Cardiomyopathy	13 (76%)	
Hypertrophic Cardiomyopathy	1 (6%)	
Viral myocarditis	1 (6%)	
L-TGA-VSD	1 (6%)	
DORV	1 (6%)	
Length of illness prior to VAD		2 weeks – 9 years
Length of hospitalization post implant (days)	42.6 (18.5)	15 days – 77 days
Time on VAD at home post discharge (days)	282.2 (268.3)	31 days – 838 days
Duration of VAD support (days)	303.1 (239.2)	62 days – 859 days
Time since transplant (days; *n=*9)	409.8 (298.0)	22 days – 961 days
Patient education		
Currently completing middle/high school	13 (76%)	
Graduated high school	2 (12%)	
Did not finish high school	1 (6%)	
No response	1 (6%)	

### Interview results

#### Discharge home on VAD

See Figure 1 for relevant quotes across thematic domains. A range of emotions were experienced when anticipating and upon initial discharge home on VAD, including excitement, hope, and nervousness. Participants stated that being on VAD support allowed them to return to enjoyable activities and be discharged from the hospital, and return to comforts of home. Discharge home on VAD support was not without challenges, as several noted that adjusting to life at home and needing to come back to the hospital for appointments, management of infections, and issues with lines and tubes were difficult. Several indicated that even though they were discharged home, they did not anticipate staying home for long, including regular back and forth travel for regular check-ups or potential unexpected admissions or complications.

**Figure 1:**
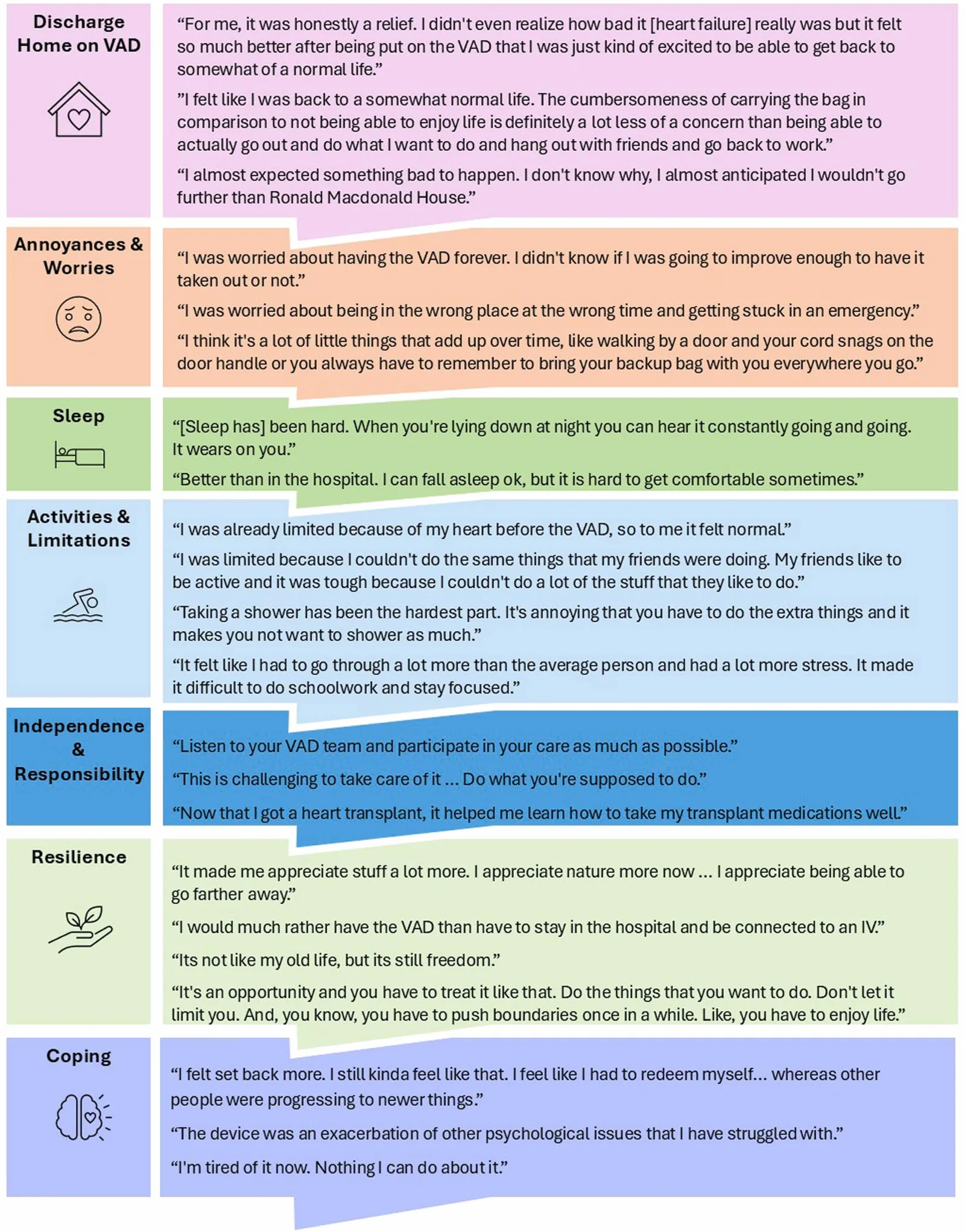
Example Participant Quotations Across Thematic Domains.

#### Annoyances and worries

Participants qualitatively reported a range of worries, including unknown length of VAD support, managing emergency situations, and needing to make sure they had batteries and supplies on outings. This is consistent with quantitative ratings and intensity of worry, such that the majority of AYAs indicated experiencing worry, along with varying intensities ranging from ‘very worried’ to ‘not at all worried’, about emergency situations (*n*=14; 82%), infections (*n*=12; 71%), bleeding (*n*=12; 71%), or device malfunction (*n*=11; 65%; see Table 2). The carrier bag was noted across AYAs as the most frequently reported item of annoyance (*n*=9; 53%), followed by length of driveline (*n*=8; 47%), driveline dressing (*n*=8; 47%), and batteries (*n*=7; 41%; see Table 2).

**Table 2:** VAD Annoyances and Worries

	*n* (%)		*n* (%)
VAD Component	Indicating annoyance or gets in the way	VAD Worries (continued)	Indicating worry
Bag	9 (53%)	Device malfunction	11 (65%)
*Specifically, bag appearance*	*3*	*Extremely worried*	*1*
*Specifically, bag weight*	*6*	*Very worried*	*2*
Length of driveline	8 (47%)	*Moderately worried*	*1*
Driveline dressing	8 (47%)	*A little worried*	*5*
Battery (size, lifespan, function)	7 (41%)	*Not at all worried*	*2*
Switching power sources	4 (24%)	Dressing changes	10 (59%)
Emergency supplies	3 (18%)	*Very worried*	*--*
Controller	2 (12%)	*Moderately worried*	*4*
Checking vital signs (e.g., doppler, BP cuff)	2 (12%)	*A little worried*	*4*
Other (i.e., alarms, location weight, adhesive)	4 (24%)	*Not at all worried*	*2*
	*n (%)*	Pain	9 (53%)
VAD Worries	Indicating worry	*Very worried*	*2*
Emergency situations	14 (82%)	*Moderately worried*	*4*
*Very worried*	*5*	*A little worried*	*2*
*Moderately worried*	*4*	*Not at all worried*	*1*
*A little worried*	*3*	Physical appearance	8 (47%)
*Not at all worried*	*2*	*Very worried*	*1*
Infection	12 (71%)	*Moderately worried*	*2*
*Extremely worried*	*2*	*A little worried*	*3*
*Very worried*	*2*	*Not at all worried*	*2*
*Moderately worried*	*2*	Neurological event (e.g., stroke)	8 (47%)
*A little worried*	*4*	*Extremely worried*	*1*
*Not at all worried*	2	*Very worried*	*--*
Bleeding	12 (71%)	*Moderately worried*	*1*
*Extremely worried*	*1*	*A little worried*	*2*
*Very worried*	*--*	*Not at all worried*	*4*
*Moderately worried*	*3*	Other (i.e., losing power, pulling	4 (24%)
*A little worried*	*7*	driveline, length of time on VAD)	
*Not at all worried*	*1*		

For some, VAD-related annoyances and worries evolved over time. One participant noted that what may have started off as smaller annoyances (e.g., lines snagging on doorknobs) eventually added up to greater difficulty coping over time, as these experiences compounded on one another. Another noted that eventually management of VAD became “second nature.” For some, increased duration on VAD support brought uncertainty, with one young adult noting they began to wonder about the “what ifs” regarding potential differences in their treatment course if things had gone differently.

#### Sleep

The majority of participants reported some difficulty sleeping (*n*=11; 65%; see Table 3). Many AYAs qualitatively noted that sleep improved naturally upon discharge home once back to the comfort of their own space and bed. While some reported that their sleep was unchanged from prior and therefore was overall generally good, others noted negative impacts due to positioning of VAD (e.g., not being able to sleep on back or preferred side), temperature of controller if under a blanket, or controller and cords falling off the bed. This was consistent with quantitative ratings on sleep difficulties (see Table 3).

**Table 3:** VAD Impacts and Limitations

Sleep Impacts	*n* (%) indicating impact
Difficulty sleeping	11 (65%)
due to… *position of VAD*	*7*
due to… *difficulty getting comfortable*	*4*
due to… *device noise/buzzing*	*3*
due to… *fear/worry about device*	*2*
due to… *waking up to use restroom*	*2*
No difficulty sleeping	6 (35%)
Activities/Limitations	*n* (%) indicating limitation
school/work participation	6 (35%)
*Extremely limited*	*--*
*Very limited*	*--*
*Moderately limited*	*2*
*A little limited*	*1*
*Not at all limited*	*3*
… play/hanging out with friends	12 (71%)
*Extremely limited*	*--*
*Very limited*	*2*
*Moderately limited*	*6*
*A little limited*	*4*
*Not at all limited*	*--*
… sports participation	10 (59%)
*Extremely limited*	*2*
*Very limited*	*5*
*Moderately limited*	*1*
*A little limited*	*1*
*Not at all limited*	*1*
… ability to move around freely	8 (47%)
*Extremely limited*	*--*
*Very limited*	*3*
*Moderately limited*	*--*
*A little limited*	*4*
*Not at all limited*	*--*
… planning for post-school plans (e.g., jobs, college)	6 (35%)
*Extremely limited*	*2*
*Very limited*	*1*
*Moderately limited*	*1*
*A little limited*	*--*
*Not at all limited*	*2*
… something else (e.g., swimming, PO intake)	6 (35%)
Responsibilities	*n* (%), *M (SD)*
How much of VAD care is your responsibility (1–10, 10=highest responsibility)?	6.7 (2.2)
Who else helps care for your VAD … *parents*	*14 (82%)*
*… siblings*	*2 (12%)*
*… other family members*	*2 (12%)*
*… someone else*	*3 (18%)*
*… no one else*	*1 (6%)*

#### Activities and limitations

All participants reported at least some limitations to activities. Quantitative ratings indicated that the majority of AYAs felt limitations in play/hanging out with friends (*n*=12; 71%), followed by sports participation (*n*=10; 59%), and ability to move around freely (*n*=8; 47%; see Table 3). Qualitative reports of limitations ranged from challenges with activities of daily living, such as showering, to engagement in preferred activities, such as swimming. Some participants identified strategies for continuing these activities (e.g., using a shower bag, assistance from caregivers in managing lines or controller while showering), while others noted that desired activities like swimming or more intense physical activity were completely restricted. Several noted that being able to work out and return to some activity was helpful in coping with VAD, noting that engaging with physical therapy and finding community on social media with those who are supported on device was helpful. For those who previously had a cardiac diagnosis which limited their ability to engage in physical activity, limitations related to VAD were not new or as impactful.

The influence of VAD on school and education was variable across participants (*n*=6; 35% indicated limitations in school/work participation and planning for post-school plans). Of those noting that VAD negatively impacted school, reasons included increased absences due to appointments or health challenges, lack of knowledge of school staff and peers regarding VAD, and overall stress of VAD impacting mental health and therefore school engagement. Qualitatively, attending school virtually was largely viewed as a positive alternative to attending in-person due to potential for negative peer attention or challenges in navigating schoolwork while managing VAD.

#### Independence and responsibility

Across interviews, participants consistently noted that managing their VAD care was at times challenging and identified that support from caregivers, family members, friends, and medical teams was critical to managing successfully. The majority of participants reported at least some support in managing VAD care at home, most of which was from parents (*n=*14; 82%; see Table 3). This was further supported by a mean rating of 6.7 (*SD=*2.2) out of 10 for how much of their VAD care was their responsibility versus others. Of note, one young adult on VAD indicated that they had no help caring for their VAD. The importance of doing what was required by VAD teams (e.g., adherence to medications, cares, appointments, etc.) and actively participating in care was underscored across interviews. Several participants noted that taking care of their medical regimen and needs related to VAD helped prepare them to be a more successful transplant recipient.

#### Resilience

Many participants noted that, despite the critical nature of being discharged home on VAD, they were able to adjust and eventually recognized being on VAD as an opportunity for continuing to live life more aligned with how they wanted to and with increased appreciation for the world around them.

#### Coping

All participants noted some difficulty coping, including managing changes in relationships, feeling different from others, and having to navigate the world in a different way. Significant coping challenges were viewed by participants as likely multi-factorial – for some, VAD stressors and coping challenges felt like an exacerbation of prior mental health concerns, while others noted that being on VAD led to perceptions that they were set back or falling behind their peers. One participant expressed feeling “medical imposter syndrome,” such that they perceive that they are not actually as critically ill as others around them and therefore shouldn’t require “special treatment.” Additionally, one young adult noted that they are overall tired of managing VAD but perceive there is nothing they can do about it now.

#### Lessons learned and words of wisdom

Among the nine participants bridged to transplant, several noted that, despite the anxiety regarding managing a VAD at home, it was “worth it” knowing that being supported on VAD allowed them to be a stronger transplant candidate. Participants provided advice for AYAs considering VAD, including finding connections via social media or other sources of community, and adapting the carrier system or bag to better align with their needs and lifestyle (e.g., finding shirts or pants with device carrying systems built in, decorating bag or carrier). Participants shared words of wisdom for other AYAs on VAD (see Figure 2 for participant quotations).

**Figure 2:**
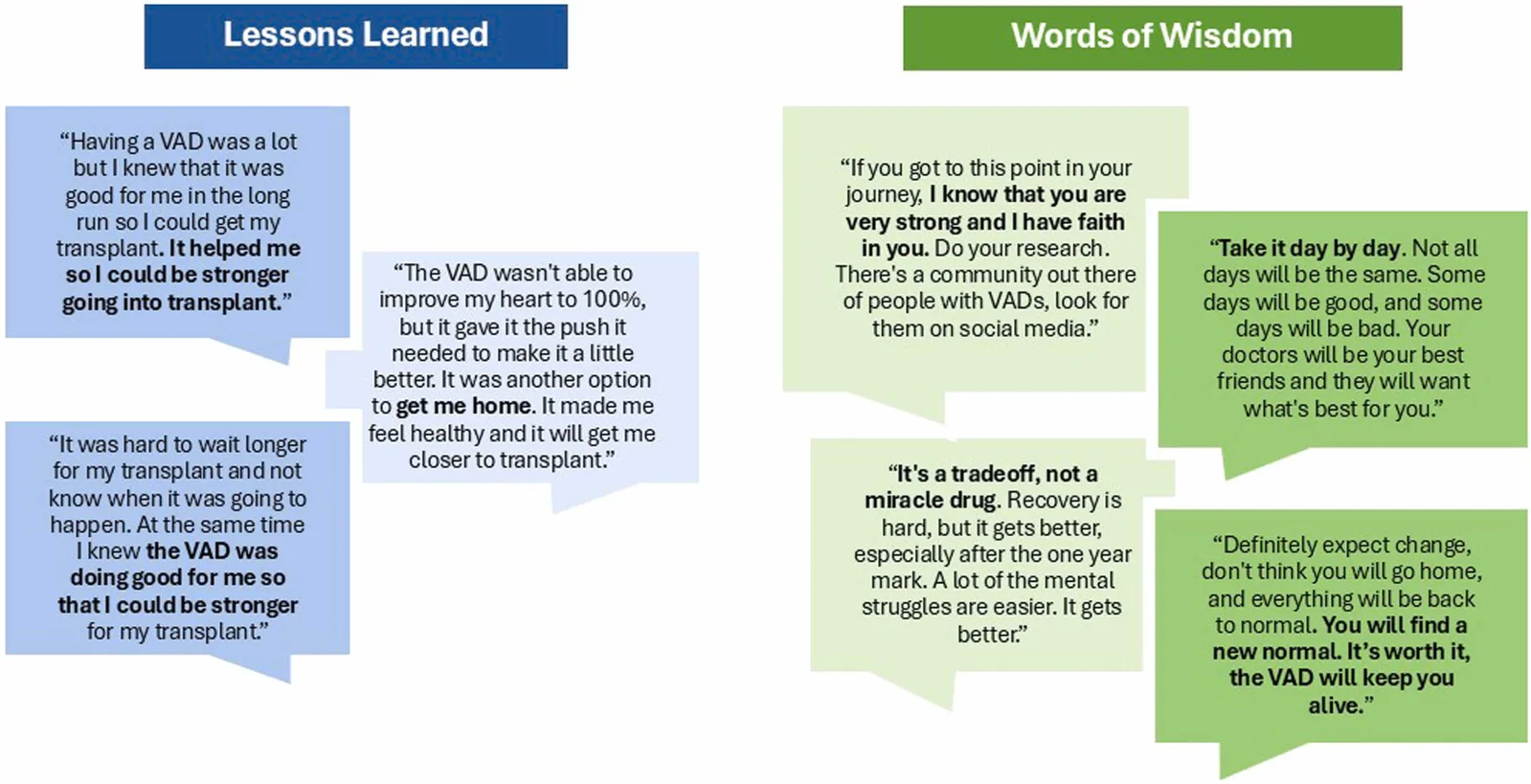
Participant Lessons Learned and Words of Wisdom.

## Discussion

The present mixed methods analysis of the AYA VAD experience highlights the complex and evolving challenges faced as outpatients. Excitement and nerves upon discharge shifted to worries about managing VAD at home and annoyances with limitations. Day-to-day annoyances and impacts on activities of daily living contributed to coping challenges, including feeling different from others and navigating shifts in relationships and independence. Despite these challenges, several AYAs reflected on their resilience in the face of medical challenges and evolved appreciation for life. These sentiments and other words of wisdom for peers considering discharge home on VAD include adapting carrying systems, finding community among others on VAD support, normalizing challenges in recovery, and taking life day by day.

Current findings are consistent with past assessments of pediatric patients supported on VAD, including overall improvements in QOL across the first 6 months post-implant,^5^ as well as shifting coping challenges over time.^6^ AYAs in the present study noted that, in general, VAD positively impacted their lives, allowing for discharge home and return to a “new normal.” However, it was not without challenges, requiring additional support from caregivers and team members, as well as connection from others on VAD, when available.

A recent consensus-based framework for the evaluation of pediatric patients for VAD candidacy reinforced recommendation for standard psychosocial screening to assess patient and family knowledge, adherence, barriers to device management, and mental health prior to VAD implantation.^15^ These screenings assess for patient and family understanding and readiness for VAD but also introduce psychosocial support services and facilitate access to potentially necessary interventions throughout VAD course. The present study highlights the importance of mental well-being and support throughout the VAD journey. Ensuring access to psychosocial support team members, such as psychology, social work, and child life, is a critical component to VAD care. As families anticipate discharge home on VAD, discharge readiness is most commonly assessed through demonstration of medical understanding, knowledge, and adherence to VAD cares.^16^ However, discharge readiness as it relates to coping and mental health is an additional component required to support patients and families, and therefore incorporation of psychosocial multidisciplinary team members in this process is warranted.^16^

The unique mental health considerations for AYAs on VAD in combination with calls for incorporation of psychosocial supports throughout the VAD experience highlights the importance of access to these supports across inpatient and outpatient settings. A recent examination across 24 centers providing VAD care found that mental and behavioral health supports vary across institutions. For example, while the majority of centers have some combination of social work, psychology, or other therapy supports (e.g., feeding, speech, occupational, physical) available while patients and families are admitted to the hospital, fewer have access to these resources upon discharge.^17^ Notably, less than half of centers indicated that routine neurodevelopmental or therapy referrals are automatically initiated by VAD teams, while others indicated these referrals are not routine or are only initiated by other subspecialty teams or families themselves.^17^ As such, timely referral opportunities may be missed or not initiated until significant challenges are present. Reasons for missed referrals in the inpatient setting may include focus from medical team members and families on the medical stability and critical nature of health status during inpatient hospitalization following VAD implant; however, as families approach discharge home on VAD, discussions about neurodevelopmental and psychosocial supports should be initiated.

Specific to mental health care, approximately 60% of centers reported outpatient therapy services available for VAD patients, while only 16% or fewer had access to group therapy or parent support groups.^17^ Given the importance of community and connection reported by AYAs in the present study, this remains an important area for growth across centers. Leveraging use of national and international learning networks across institutions is one avenue through which patients and families may find connection and individual institutions may find additional peer support opportunities. Given the likely low patient volume within centers supporting pediatric patients at home on VAD, this calls for collaboration across institutions to explore ways to promote connection and peer support for patients who desire these resources, leveraging use of technology, virtual programming, and social media opportunities to promote connection across geographical regions.

Integrated psychosocial support opportunities for pediatric patients and families on VAD are needed, particularly in the outpatient setting where these services are not as readily available. Institutions supporting patients on VAD should advocate for integration of psychosocial support providers within their VAD teams and structure the VAD process to allow opportunity to meet these providers and initiate timely services as medically able and indicated. Such opportunities may include standardized psychosocial screening and evaluations prior to VAD implantation as part of routine VAD evaluation and assessment of coping and need for psychosocial supports in discharge readiness. Additionally, a review of patient-facing VAD informational resources found that while all materials referenced potential benefits, fewer discussed lifestyle considerations,^18^ several of which were prominently featured in AYA interviews (e.g., sleep challenges, limitations on outings, difficulty with activities of daily living, return to school). VAD centers should consider what resources and materials are shared with their AYA patients and families and ensure a balanced approach to benefits and lifestyle challenges is reflected. Quality improvement initiatives would be valuable to determine whether development of materials such as these and other psychosocial support efforts (e.g., support groups) improve patient and family coping upon discharge or improve timely access to necessary interventions, such as establishing psychotherapy services outside of the hospital. Lastly, as advances in VAD technology continue, along with increased use of VAD as a destination therapy, patient-facing quality of life factors should be considered in updating and developing devices which allow for improved patient experience (e.g., sleep disturbance).

The preliminary findings of this investigation must be considered in light of several study limitations, including this single sample and generalizability across settings and centers, as well as patient experience, including variability in length of time on VAD, being on VAD at time of interview, and variation in experience of explant to transplant. Further, while a young adult participated in review and feedback of the interview guide, additional inclusion of the adolescent perspective would have potentially given additional insight during guide development. Future research should further parse apart the AYA experience during and post-VAD support, as well as across those bridged to recovery versus transplant. Longitudinal examination of AYA experience would provide additional insights into changes in coping and areas of support over time, as well as explore potential temporal or causal links between mental health concerns and VAD implant. Lastly, while participants not discharged home on VAD were excluded from the present study, additional research related to the AYA experience for those in the hospital on VAD support is warranted to ensure appropriate supports are available for all.

## Conclusion

Significant life disruptions and coping challenges are prevalent among AYAs discharged home on VAD, including navigating activity limitations, decreased independence, and worries regarding device. However, AYAs also report resilience and increased gratitude for life and the opportunity their VADs provided. Given the complexity of coping experiences prevalent in this unique period of development and medical state, robust psychosocial supports for AYAs and families are indicated and should be available to patients and families in inpatient and outpatient settings. Efforts to build community support for outpatients on device, including in-person and online AYA peer and caregiver supports, should be evaluated for efficacy in improving coping and the outpatient VAD experience as a whole.

## Funding Statement

There are no funding sources to declare.

## Conflict of Interest Statement

The authors declare no disclosures or conflicts of interest relevant to this manuscript.

## Declaration of Competing Interest

The authors declare that they have no known competing financial interests or personal relationships that could have appeared to influence the work reported in this paper.
