# Supplementary material for: “It’s Not Like My Old Life, But It’s Still Freedom”: An ACTION learning network mixed methods analysis of the adolescent ventricular assist device experience at home
Source: JHLT Open. 2026 Sep 16;14:100685. doi: 10.1016/j.jhlto.2026.100685 (PMC13656947; doi:10.1016/j.jhlto.2026.100685)
Supplement: Supplementary file 1 [file mmc1.docx]

**Appendix A. Final Semi-structured Interview Guide**

**Semi-Structured Interview Guide**

(to be completed by Fellow with patient, transcribe answers verbatim, as able)

Today I’ll be asking you questions about your experience as a teenager/young adult on VAD living at home. Some of the questions will have multiple choice options and some questions will ask you to describe your experience in your own words. There are no right or wrong answers to any of these questions, we just want to know your experience! I’ll be writing down your responses as we go along. What questions do you have before we get started?

**Domain 1: Discharge Home on VAD**

1A. First, I want you to try to think back to when you were in the hospital about to be discharged home on VAD support – what did that feel like for you/what was going through your mind?

__________________________________________________________________________________

__________________________________________________________________________________

__________________________________________________________________________________

__________________________________________________________________________________

1B. What things were you excited about?

__________________________________________________________________________________

__________________________________________________________________________________

1C. What things were you nervous about?

__________________________________________________________________________________

__________________________________________________________________________________

1D. What has gone well at home on VAD support?

__________________________________________________________________________________

__________________________________________________________________________________

1E. What has been hard at home on VAD support?

__________________________________________________________________________________

__________________________________________________________________________________

**Domain 2: Annoyances/Worries**

2A. As you know, VADs have lots of different parts and pieces - what, if any, of these parts have you felt have gotten in the way of things or annoyed you at times?

☐ Driveline dressing	☐ Length of driveline

☐ Controller	☐ Switching power sources (e.g., going from wall to battery)

☐ Battery (size, lifespan, function)	☐ Checking vital signs (e.g., doppler/manual BP cuff)
☐ Emergency supplies	☐ Bag (including straps etc.)

☐ Something else: ________	[ ] Appearance of bag?

[ ] Weight of bag?

2B. What changes, if any, have you made to manage these annoyances? *(possible probing statements might include: some teenagers have to rearrange their rooms so they are closer to an outlet, getting a different bag or carrying system for their VAD and parts, etc.)*

__________________________________________________________________________________

__________________________________________________________________________________

☐ No changes/adaptations made

2C. There are multiple different things that teenagers/young adults could worry about while living on VAD - what, if any of these, have you felt worried about (*Note: if endorsed, complete Likert scale*):

□	Dressing changes

□ 1=not at all worried, □ 2=a little worried, □ 3=moderately worried, □ 4=very worried, □ 5=extremely worried

□	Emergency situations

□ 1=not at all worried, □ 2=a little worried, □ 3=moderately worried, □ 4=very worried, □ 5=extremely worried

□	Physical appearance on VAD

□ 1=not at all worried, □ 2=a little worried, □ 3=moderately worried, □ 4=very worried, □ 5=extremely worried

□	Device malfunction

□ 1=not at all worried, □ 2=a little worried, □ 3=moderately worried, □ 4=very worried, □ 5=extremely worried

□	Infection

□ 1=not at all worried, □ 2=a little worried, □ 3=moderately worried, □ 4=very worried, □ 5=extremely worried

□	Bleeding

□ 1=not at all worried, □ 2=a little worried, □ 3=moderately worried, □ 4=very worried, □ 5=extremely worried

□	Neurological event, like a stroke

□ 1=not at all worried, □ 2=a little worried, □ 3=moderately worried, □ 4=very worried, □ 5=extremely worried

□	Pain

□ 1=not at all worried, □ 2=a little worried, □ 3=moderately worried, □ 4=very worried, □ 5=extremely worried

□	Something else: _______________________________________________________________

□ 1=not at all worried, □ 2=a little worried, □ 3=moderately worried, □ 4=very worried, □ 5=extremely worried

2D. Tell me a little more about these worries *(possible probing questions: what do these worries look or feel like, does the worry get in the way of anything?):*

__________________________________________________________________________________

__________________________________________________________________________________

__________________________________________________________________________________

__________________________________________________________________________________

**Domain 3: Sleep**

3A. Let’s talk about sleep! Since being on VAD, how would you say your sleep has been?

__________________________________________________________________________________

__________________________________________________________________________________

__________________________________________________________________________________

3B. There are lots of different ways people’s sleep can be impacted:

□	Would you say you have difficulty falling asleep?

□	Staying asleep?

□	Waking up in the middle of the night?

□	When you wake up in the morning, do you feel rested?

□	Something else: ______________________________________________________

□	No difficulty sleeping

3C. If having difficulty, what things about the VAD have impacted your sleep:

□	Position of VAD

□	Difficulty getting comfortable

□	Device noise/buzzing

□	Fear/worry about device

□	Something else: _______________________________________________________

3D. Anything else with sleep since being on VAD support that I have not asked about?

__________________________________________________________________________________

__________________________________________________________________________________

**Domain 4: Activities/Limitations**

4A. Sometimes being on VAD support can feel limiting to what activities you can do, does that feel true for you?

__________________________________________________________________________________

4B. If yes, what activities have felt limited? (*Note: if endorsed, complete Likert scale rating*):

□	School/work participation

□ 1=not at all limited, □ 2=a little limited, □ 3=moderately limited, □ 4=very limited, □ 5=extremely limited

□	Play/hanging out with friends

□ 1=not at all limited, □ 2=a little limited, □ 3=moderately limited, □ 4=very limited, □ 5=extremely limited

□	Sports participation

□ 1=not at all limited, □ 2=a little limited, □ 3=moderately limited, □ 4=very limited, □ 5=extremely limited

□	Ability to move around freely

□ 1=not at all limited, □ 2=a little limited, □ 3=moderately limited, □ 4=very limited, □ 5=extremely limited

□	Planning for post-school plans (e.g., jobs, college applications, etc.)

□ 1=not at all limited, □ 2=a little limited, □ 3=moderately limited, □ 4=very limited, □ 5=extremely limited

□	Something else: ____________________________________________________________

□ 1=not at all limited, □ 2=a little limited, □ 3=moderately limited, □ 4=very limited, □ 5=extremely limited

□	No perceived limitations

4C. In what ways has this been limiting for you?

__________________________________________________________________________________

__________________________________________________________________________________

__________________________________________________________________________________

__________________________________________________________________________________

4D. Are you going to school/work now/were you going to school/work when you were on VAD?

□	Yes	□	No *(If no, skip to Domain 5)*

4E. If applicable, when you were going to school on VAD, were you participating in gym class?

□	Yes	□	No

4F. If applicable, when you were going to school, were you participating in extracurriculars or clubs?

□	Yes	□	No

4G. If yes, what has/was attending school/work on VAD been like for you? *(possible probing questions: are there things that were easier or harder than you expected compared to going to school before having VAD?)*

__________________________________________________________________________________

__________________________________________________________________________________

__________________________________________________________________________________

__________________________________________________________________________________

4H. Is there anything that surprised you about going back to school/work?

__________________________________________________________________________________

__________________________________________________________________________________

__________________________________________________________________________________

__________________________________________________________________________________

**Domain 5: Independence/Responsibility**

5A. On a scale of 1 to 10 with 1 being no responsibility, and 10 being full responsibility, how much of your VAD care is your responsibility?

1	2	3	4	5	6	7	8	9	10

5B. Oftentimes caring for a VAD is a team effort. What things are *you* responsible for in managing your VAD and overall health?

__________________________________________________________________________________

__________________________________________________________________________________

__________________________________________________________________________________

__________________________________________________________________________________

5C. Who else helps care for your VAD?

- My parent(s)
- My sibling(s)
- Other family members
- My friends
- Someone else: _____________________________________________________
- No one else

5D. What things are *they* responsible for in managing your VAD and overall health?

__________________________________________________________________________________

__________________________________________________________________________________

__________________________________________________________________________________

__________________________________________________________________________________

5E. Who do you feel worries the most about your VAD?

- You
- My parent(s)/caregiver(s)
- My friend(s)
- My doctor(s)
- Someone else: _____________________________________________________
- No one worries about my VAD

5F. Why do you think that is? *(include brief summary of prior response, e.g., you worry more than your parents, your parents worry more than you, etc.)*

__________________________________________________________________________________

__________________________________________________________________________________

__________________________________________________________________________________

__________________________________________________________________________________

**Domain 6: Resilience**

6A. What, if any, positive things have you experienced because you have a VAD?

__________________________________________________________________________________

__________________________________________________________________________________

__________________________________________________________________________________

6B. What, if any, negative things have you experienced because you have a VAD?

__________________________________________________________________________________

__________________________________________________________________________________

__________________________________________________________________________________

**Domain 7: Coping**

7A. There are lots of different ways people can cope with challenging things – what are some of the ways you cope with being on VAD?

__________________________________________________________________________________

__________________________________________________________________________________

__________________________________________________________________________________

7B. Are there ways that you have felt different from your peers?

__________________________________________________________________________________

__________________________________________________________________________________

__________________________________________________________________________________

**Domain 8: Lessons Learned/Words of Wisdom**

8A. Is there anything you wish you knew before starting on VAD?

__________________________________________________________________________________

__________________________________________________________________________________

__________________________________________________________________________________

8B. What would you tell other teenagers considering having a VAD placed/going home on VAD?

__________________________________________________________________________________

__________________________________________________________________________________

__________________________________________________________________________________

8C. Anything else that we have not talked about or that I have not asked that you would want to share about your experience on VAD?

__________________________________________________________________________________

__________________________________________________________________________________

__________________________________________________________________________________
